# Cost-Effectiveness of Aerial Logistics for Maternal and Newborn Health: A Simulation-Based Analysis Grounded in Real-World Evidence from the Ashanti Region in Ghana

**DOI:** 10.36469/001c.143065

**Published:** 2025-09-17

**Authors:** Maria J. Ospina-Fadul, Pedro Kremer, Florence Haruna, Fred Adomako-Boateng, Kenneth Fosu Oteng, Diana N. Tsali

**Affiliations:** 1 Monitoring & Evaluation Zipline International, South San Francisco, California; 2 Monitoring & Evaluation Zipline International, Philadelphia, Pennsylvania; 3 Monitoring & Evaluation Zipline International, Accra, Ghana; 4 Ashanti Regional Health Directorate, Ghana Health Service, Kumasi, Ghana; 5 Kumasi South Hospital, Ghana Health Service, Kumasi, Ghana

**Keywords:** aerial logistics, cost-effectiveness, maternal health, newborn health, zero-dose children, last-mile delivery

## Abstract

**Background:**

In sub-Saharan Africa, low antenatal care (ANC) coverage and limited access to facility-based deliveries remain key drivers of adverse maternal and newborn health (MNH) outcomes. Inadequate service provision at health facilities and insufficient care-seeking behavior are exacerbated by supply chain inefficiencies that restrict access to essential maternal health commodities. Aerial logistics (centralized storage and drone delivery) has shown promise as a novel approach to addressing these logistical challenges and supporting maternal health service delivery, but its cost-effectiveness has not been evaluated.

**Objectives:**

This study evaluates the cost-effectiveness of aerial logistics as an intervention for MNH. It builds on previously observed programmatic effects (increases in ANC visits, facility-based deliveries, and reductions in maternal mortality in Ghana’s Ashanti Region) to model downstream health outcomes and estimate incremental economic value.

**Methods:**

Using microsimulation and published epidemiological parameters, the study models additional health outcomes resulting from increased service utilization among 11 249 pregnant women, including reductions in low birth weight, postpartum hemorrhage, neonatal mortality, and early-onset neonatal sepsis. Alongside the observed maternal mortality reduction, all outcomes are translated into life-years saved and discounted disability-adjusted life-years (DALYs) averted. Cost estimates are based on real-world aerial logistics operations and national data on health system expenditures and household out-of-pocket costs. Incremental cost-effectiveness ratios (ICERs) are calculated from both health system and societal perspectives. Uncertainty is addressed through one-way and probabilistic sensitivity analyses.

**Results:**

The intervention averted 3754.99 discounted DALYs at a net cost of US 400 987fromthegovernmentperspective,yieldinganICERofUS106.79 per DALY averted. From the societal perspective, the ICER was US 377.82.ThecostperprematuredeathavertedwasUS3072.87. Service utilization ICERs included US 88.46peradditionalANCuser,US2.24 per ANC visit, and US $5.60 per facility-based delivery. All estimates remained below national cost-effectiveness thresholds across sensitivity analyses.

**Discussion:**

Aerial logistics yields substantial health and economic gains, derived from previously documented increases in service utilization, and ranks among the most cost-effective documented MNH interventions.

**Conclusions:**

Aerial logistics is a highly cost-effective, scalable strategy to improve maternal and newborn health in resource-limited settings.

## BACKGROUND

Low antenatal care (ANC) attendance and low rates of facility-based deliveries remain major contributors to poor maternal and neonatal health (MNH) outcomes in sub-Saharan Africa. Limited engagement with routine ANC reduces opportunities for early detection of complications and weakens birth preparedness, while delivering outside of health facilities increases the risk of adverse outcomes from preventable causes such as postpartum hemorrhage, sepsis, and obstructed labor.^1^ These service gaps are consistently associated with higher rates of maternal and newborn morbidity and mortality. Moreover, inadequate maternal care contributes to the incidence of low birth weight (LBW), which is linked to both neonatal mortality and long-term health risks.^2^

While ANC and institutional deliveries are well-established as cost-effective strategies to improve maternal and neonatal health, limited healthcare budgets in many African settings necessitate careful assessment of interventions aiming to increase the utilization of these services and the corresponding health outcomes. Basic maternal health packages, comprising ANC, skilled birth attendance, and postpartum care, typically cost between US $150 and US $1000 per disability-adjusted life-year (DALY) averted in sub-Saharan Africa, with community-based strategies such as women’s groups or health worker training falling at the lower end. More intensive interventions, like increased availability of emergency cesarean sections for obstructed labor, show greater variation in cost-effectiveness (US $200-$4000 per DALY) depending on the context.^3,4^ Interventions aimed at increasing ANC utilization have also shown wide cost ranges, with some studies estimating the cost per additional ANC visit at US $5 to $30.^5^ However, long-term benefit estimation remains difficult due to variations in health system capacity, intervention design, and lifetime health outcome modeling.These complexities highlight the need for innovative, rigorously evaluated approaches to improve maternal health service uptake.

One such innovation gaining traction is the use of unmanned aerial vehicles, or drones, as a means of addressing persistent logistical barriers in maternal healthcare delivery. In many parts of sub-Saharan Africa, rural clinics face chronic stockouts of essential maternal health commodities due to weak infrastructure and transportation inefficiencies, which compromise timely access to life-saving care. Drone delivery networks offer a potentially transformative solution by enabling on-demand transport of blood, vaccines, and essential medicines, thereby improving supply chain responsiveness and reliability.^6^ While existing research has primarily focused on the use of drones for immunization programs and emergency blood deliveries (areas characterized by urgent cold chain and time-sensitive needs) a growing body of evidence is beginning to explore their broader impact on maternal health.

Notably, a recent evaluation of Zipline’s aerial logistics program in Ghana’s Ashanti Region (2017-2022) provides the first large-scale evidence linking drone-enabled delivery systems to improvements in maternal programmatic and health outcomes beyond emergency response. Zipline, operational in Ghana since 2018, delivers blood, vaccines, and medicines to rural facilities, addressing the delays common in traditional last-mile delivery (LMD), which relies on sporadic truck shipments and motorbike deliveries. The study found that facilities served by drones saw a statistically significant 19.9% increase in ANC attendance and a 25% rise in facility-based deliveries, while reducing maternal mortality by 56.4%. By ensuring rapid, on-demand access to life-saving supplies, drones significantly enhanced maternal health services and patient confidence in healthcare availability.

Because there is a critical need to evaluate not only the clinical impact but also the economic efficiency of such interventions, the present study builds on this impact evaluation to assess the cost-effectiveness of aerial logistics as a MNH intervention in Ghana. Drawing on real-world cost data and microsimulated, modeled health outcomes, anchored in primary impact data from a previous study,^7^ the analysis estimates the cost per additional ANC visit, cost per DALY averted, and cost per maternal death averted. These findings aim to inform policymakers in low- and middle- income countries (LMICs) on the potential scalability and economic viability of drone-enabled supply chains as a strategy to strengthen maternal and newborn healthcare delivery.

## METHODS

This analysis evaluates the cost-effectiveness and health impact of expanding aerial logistics for maternal and neonatal care in Ghana. Incremental cost-effectiveness ratios (ICERs) are estimated for 5 outcomes: cost per additional ANC user, cost per additional ANC visit, cost per premature death averted, cost per life year saved (LYS), and cost per DALY averted, from both societal and health system perspectives.

In addition to maternal deaths averted, the analysis incorporates direct programmatic results and modeled estimates of key maternal and neonatal health outcomes, including reductions in LBW, postpartum hemorrhage (PPH), neonatal mortality, and neonatal sepsis. These outcomes are driven by increased ANC attendance and facility-based deliveries attributed to the intervention. The specific modeling approaches for each impact pathway are detailed in subsequent sections. Cost inputs reflect both public healthcare expenditures and household out-of-pocket (OOP) payments, and health benefits are translated into DALYs using standard burden of disease methodologies.

To account for uncertainty in literature-derived parameters, one-way sensitivity analyses were conducted and visualized using a tornado diagram to identify key cost-effectiveness drivers. Additionally, probabilistic sensitivity analysis using Monte Carlo simulation was employed to generate cost-effectiveness planes and acceptability curves. All analyses were performed in R (version 4.3.2).

### Incremental Cost-Effectiveness Ratios

Pursuing a comparison with available ICERs for interventions in the same health area, we estimated several ICERs: cost per additional ANC user and per additional ANC visit; cost per death averted; and cost per averted DALY, which in this case equates to cost per life-year saved (LYS). The latter ICER was estimated from both the societal and health system perspectives. All costs were reported in 2024 US dollars. Cost per DALY averted, from both health system and societal perspectives, was calculated using the equations below, with details on each component provided in the health impact and cost sections.

**Figure:**
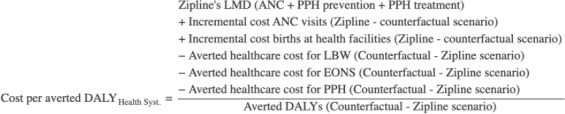


**Figure:**
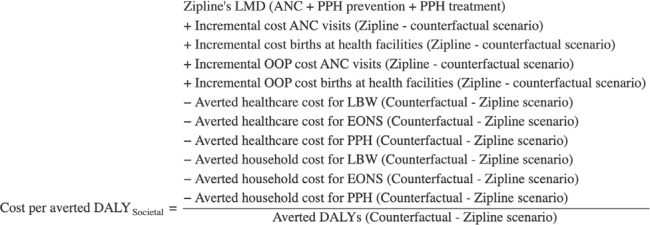


### Population and Health Outcomes Considered

Our study took into account 1 directly measured health impact—the reduction in maternal mortality—and 4 health impacts that were estimated through modeling: the reduction in low LBW, the reduction in PPH incidence, the reduction in neonatal mortality, and the reduction in neonatal sepsis. To estimate the reduction in LBW prevalence and the reduction in PPH incidence, we relied on the increased ANC visits, particularly focusing on 2 key thresholds: the proportion of women reaching at least 8 ANC visits and the proportion of women moving from zero to at least 1 ANC visit. Literature in Africa, and in Ghana in particular, shows that the first threshold is significantly associated with improved neonatal health outcomes,^8^ while the second is significantly linked to a reduced incidence of PPH.^9^ To model the reduction in neonatal mortality and neonatal sepsis, we used the increase in deliveries at health facilities as the primary variable. This approach is supported by existing literature, which demonstrates a significant association between increased deliveries at health facilities—as opposed to home deliveries— in low-income settings and both reduced neonatal mortality^10^ and a lower incidence of neonatal sepsis.^11^

**Table 1** presents the main parameters used in our analysis, as well as the estimated parameters derived using the methodology described for each case in terms of averted burden of disease.

**Table 1.** Input Parameters and Modeled Outputs for the Health Outcomes

**Parameter**	**Value**	**Source**
Relative risk of neonatal mortality for health facility delivery compared with home delivery	0.71	Tura et al (2013)
Baseline neonatal mortality rate per 1000 births	17	DHS 2023
Additional births at health facilities due to intervention	14 619.63	Estimated/model output
Averted deaths of neonates due to delivery at health facility	72.07	Estimated/model output
Discount rate	0.05	Set parameter
Prevalence of LBW	0.11	DHS 2023
OR of LBW with <8 ANC	0.24	Adjei-Gyamfi et al (2023)
Total LBW cases (2021-2022)	1207.11	Estimated/model output
Overall odds of LBW	0.12	Estimated/model output
Adjusted odds for ≥8 ANC visits	0.03	Estimated/model output
Prevalence for mothers with ≥8 ANC visits	0.03	Estimated/model output
No. of LBW babies for mothers with ≥8 ANC visits	106.89	Estimated/model output
No. of LBW cases for mothers with <8 ANC visits	1100.22	Estimated/model output
Prevalence for mothers with <8 ANC visits	0.15	Estimated/model output
Net reduction in LBW with intervention	270.04	Estimated/model output
Proportion of moderate LBW cases	0.78	DHS 2023
Proportion of very LBW cases	0.14	DHS 2023
Proportion of extremely LBW cases	0.08	DHS 2023
Averted moderate LBW cases	210.00	Estimated/model output
Averted very LBW cases	38.24	Estimated/model output
Averted extremely LBW cases	21.79	Estimated/model output
Neonatal mortality rate (reference group)	0.007453	O’Leary et al (2017)
Early infancy mortality rate (reference group)	0.008182	O’Leary et al (2017)
Late infancy mortality rate (reference group)	0.006936	O’Leary et al (2017)
Total neonatal deaths averted (low birthweight, all groups)	22.20	Estimated/model output
Incidence of PPH among all deliveries	0.199	Bautista et al (2024)
Proportion of PPH that are severe	0.248	Bautista et al (2024)
Odds ratio of PPH for women with 0 ANC vs ≥1 ANC	3.43	Moyo et al (2024)
PPH incidence among women with ≥1 ANC	0.19	Estimated/model output
PPH incidence among women with 0 ANC	0.44	Estimated/model output
Reduction in women with 0 ANC visits due to intervention	363.56	Estimated/model output
Reduction in PPH cases due to ANC coverage increase	92.78	Estimated/model output
Reduction in severe PPH cases due to ANC coverage increase	23.03	Estimated/model output
Proportion of deliveries that are vaginal	0.85	DHS 2022 (rural)
Maternal deaths (2021-2022) observed in Zipline served facilities	28.00	GHS
Mortality reduction impact from Zipline	0.56	Kremer et al (2025)
Maternal deaths (2021-2022) counterfactual	64.22	Estimated/model output

Abbreviations: ANC, antenatal care; DALY, disability-adjusted life-year; LMD, last-mile delivery; NHIS, National Health Insurance Scheme; OOP, out of pocket; PPH, postpartum hemorrhage.

### Statistical and Modeling Approach for Estimating Distribution of Additional ANC Visits

To estimate changes in ANC visit distribution and the number of additional women crossing these thresholds, we used a probabilistic microsimulation approach that applies stochastic resampling to model shifts in ANC visit patterns following the introduction of Zipline’s aerial logistics. The first step in this approach was estimating the target population: pregnant women who would have Zipline-served health facilities as their reference facility. While we had data on the number of births at these facilities, birth data alone does not fully represent the ANC-seeking population, as both referral hospitals and primary health centers serve overlapping catchment areas. Instead, we used the 2021 and 2022 birth cohorts from the 2023 Demographic and Health Survey (DHS) in Ghana to estimate the distribution of pregnant women across ANC visit categories. We first estimated the counterfactual number of ANC visits, which represents what would have been observed in Zipline-served facilities in the absence of aerial logistics. Given that the observed number of ANC visits was 44 384 in 2021 and 42 095 in 2022, and that aerial logistics is associated with a 19.9% increase in ANC visits, we adjusted these figures by removing this effect, resulting in an estimated counterfactual number of ANC visits of 35 533 for 2021 and 33 703 for 2022. These adjusted visit numbers were then distributed in the number of ANC visit categories according to the national ANC visit distribution observed in the corresponding Demographic Health Survey (DHS) birth cohorts, allowing us to estimate the most likely target population size, which was 5703 pregnant women in 2021 and 5546 in 2022.

For simplicity, we worked with a single target population by aggregating the 2021 and 2022 distributions, summing the number of women per ANC visit category. We then conducted 1000 probabilistic microsimulations to model how ANC visit distributions changed after the observed increase in ANC visits due to aerial logistics. In each simulation, a woman was randomly selected, and additional ANC visits were assigned based on conditional probabilities derived from her baseline ANC category. The assumption that conditional probabilities remain valid after the increase (ie, the ANC distribution shape remains statistically unchanged) is supported by the pairwise Wilcoxon test and the Kolmogorov- Smirnov test, which found no significant differences between the 2017 ANC distribution (from the Multiple Indicator Cluster Survey for Ghana) and the 2020-2021 birth cohorts ANC distributions (from DHS), even after Bonferroni correction. This process was repeated iteratively until the total number of additional ANC visits observed due to aerial logistics was fully allocated. This approach ensures that the total target population remains unchanged while capturing the redistribution of ANC visit categories.

**Figure 1.**
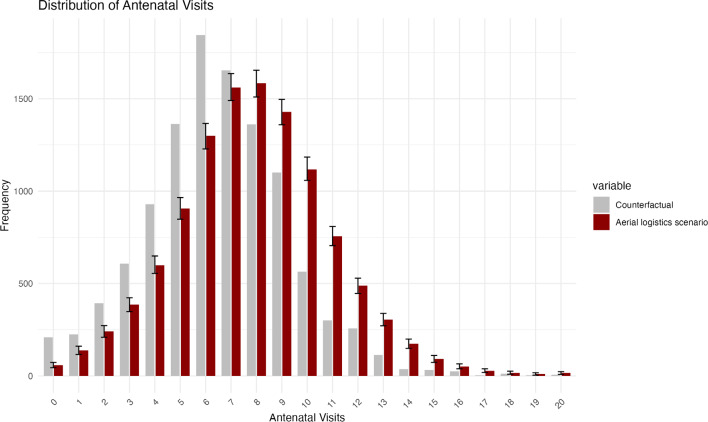
Estimated Distribution of Antenatal Care Visits in the Aerial Logistics and Counterfactual Scenarios This figure shows the estimated number of pregnant women by ANC visit count in 2 scenarios: without aerial logistics (gray) and with aerial logistics (red), based on a probabilistic microsimulation. The simulation draws from the 2021-2022 DHS birth cohorts in Ghana and uses the19.9% increase in ANC visits due to Zipline’s aerial logistics from Kremer et al (2025). Total ANC visits were first adjusted to remove the observed effect of aerial logistics and then redistributed across visit categories using national DHS patterns. The red bars reflect 1000 stochastic simulations reallocating visits within the same target population, based on conditional probabilities of visit increases. Confidence intervals (black lines) are shown for the aerial logistics scenario.

The counterfactual and Zipline-adjusted ANC visit distributions, along with confidence intervals for the latter, are presented in **Figure 1**, illustrating both the increase in ANC utilization and the redistribution across visit categories resulting from aerial logistics. Based on these distributions, we estimated that the intervention enabled approximately 2252 additional women to reach the threshold of 8 or more ANC visits, and 363 additional women to transition from no ANC attendance to at least 1 visit.

### Health Outcome Estimation Methods

**Reduced prevalence of LBW and PPH**: We used an odds ratio (OR) and observed incidence approach to model reductions in both LBW prevalence and PPH incidence because of increased ANC utilization. This method applies ORs from the literature, the observed incidence, and the segmentation of a given population in ANC visit categories, to estimate the incidence of these conditions in different ANC visit categories and then projects how changes in ANC distribution impact overall incidence.

For LBW, we used an OR of 0.24 for 8 or more ANC visits compared with 7 or fewer, based on literature from Ghana.^8^ Using this OR, we estimated the probability of LBW among women attending 8 or more ANC visits and applied this probability to the number of women shifting into this category due to increased ANC utilization. This allowed us to estimate the reduction in overall LBW rates. To allocate the additional LBW cases into the 3 standard LBW categories, we followed the classification used in previous studies,^12,13^ which define birth weight thresholds as LBW between 2.00 to 2.49 kg, very low birth weight (VLBW) between 1.50 and 1.99 kg, and extremely low birth weight (ELBW) as less than 1.50 kg. We assumed that the distribution of cases across these 3 categories follows the proportions recorded in the 2023 DHS.

For PPH, we followed the same approach using an OR of 3.43 for 0 visits vs 1 or more ANC visits, based on a systematic review.^9^ This OR reflects the significantly higher likelihood of PPH among women who receive no ANC service compared with those attending at least 1 visit. By applying this ratio to the group incidence of PPH for women with 0 vs at least 1 ANC visit, and considering the number of women moving from the first group to the latter, we estimated the reduction in PPH incidence attributable to improved ANC access.

**Reduced mortality due to reduction in LBW prevalence**: To estimate the impact of LBW on neonatal and infant mortality, we used a hazard ratio-based mortality estimation approach. A previous study^13^ estimated hazard ratios (HRs) for mortality among infants in 3 birth weight categories—2.00 to 2.49 kg, 1.50 to 1.99 kg, and less than 1.50 kg—compared with non-LBW infants across the neonatal, early infancy, and late infancy periods in Ghana. We applied these hazard ratios to estimate the overall reduction in mortality resulting from the decrease in LBW incidence due to improved ANC utilization.

**Reduced neonatal mortality due to increased number of deliveries at health facilities**: To estimate the impact of increased health facility deliveries on neonatal mortality, we used a relative risk (RR)–based mortality estimation approach. A systematic review^10^ estimated an RR of 0.71 (95% CI: 0.54-0.87) for neonatal mortality among births occurring in health facilities compared with non-facility settings in sub-Saharan Africa. We applied this RR to estimate the reduction in neonatal deaths resulting from the increase in facility deliveries attributed to the intervention. Specifically, we calculated the number of additional facility births using the observed births at Zipline-served health facilities and multiplied by the baseline neonatal mortality rate for Ghana and the proportional reduction in risk associated with facility-based delivery.

**Reduced neonatal early-onset sepsis**: To estimate the impact of increasing health facility deliveries on neonatal sepsis incidence, we applied a risk reconstruction approach using population-weighted averages and an OR to represent the differential risk. In estimating the national incidence of early-onset neonatal sepsis (EONS) in Ghana, we drew on published incidence data from low- and middle-income countries, where the incidence of neonatal sepsis in the overall time frame has been reported as 2824 cases per 100 000 live births (95% CI: 1892-4194).^11^ As early-onset cases typically represent approximately half of all neonatal sepsis cases in Ghana,^14^ this proportion yielded a conservative estimated incidence of 1412 EONS cases per 100 000 live births (95% CI: 946-2097). This estimate provided a contextually appropriate benchmark for Ghana in the absence of nationally representative surveillance data. According to the Ghana 2022 DHS, 86% of births occur in health facilities, while 13% occur at home. A 2023 study from Ghana reports an OR of 3.6 (95% CI: 1.83-7.05) for neonatal sepsis in home births compared to facility births. Using these parameters, we algebraically approximated the underlying incidence rates for each setting, estimating the sepsis risk at 0.0048 in facilities and 0.0203 in home settings. We then calculated the number of cases averted by shifting a given number of births from home to facility deliveries, multiplying the difference in risk by the number of shifted births.

**DALY and LYS estimation**: Averted DALYs from this intervention only accounted for LYS from reduced maternal mortality and infant mortality in the early infancy and late infancy periods (corresponding to reduce LBW burden) and in the neonatal period (corresponding to both the reduction in LBW burden and the increase in deliveries at health facilities) and did not include years of life with disability corresponding to hysterectomies or long-term health consequences of LBW. All DALYs displayed in the results were discounted at an annual rate of 3%.

### Costs

This economic evaluation was conducted from both government and societal perspectives. The government perspective included costs borne by the public health system, such as treatment costs covered by the Ghanaian National Health Insurance Scheme (NHIS) and the incremental costs of aerial logistics. The societal perspective incorporated both the government costs and additional OOP expenditures by households, as well as indirect nonmedical costs such as wage losses and transportation incurred by caregivers. Costs were grouped into 3 main categories: averted costs from reduced disease burden, increased utilization of maternal health services, and logistics-related delivery costs.

**Table 2** summarizes the key cost-related input parameters and the corresponding modeled outputs used in our analysis. It includes estimates of costs and savings from both the healthcare system and societal perspectives, derived using the methodology outlined for each intervention and associated outcome.

**Table 2.** Input Parameters and Modeled Outputs for the Healthcare and Societal Costs and Savings (2024 US Dollars)

**Parameter**	**Value ($)**	**Source**
OOP cost per mild PPH case	99.50	Bautista et al (2024)
OOP cost per severe PPH case	668.00	Bautista et al (2024)
Health system cost per mild PPH case	72.90	Bautista et al (2024)
Health system cost per severe PPH case	344.35	Bautista et al (2024)
Total OOP cost averted due to PPH reduction	22 322.21	Estimated/model output
Total NHIS cost averted due to PPH reduction	13 014.14	Estimated/model output
Health system cost per neonatal sepsis case	404.60	Okomo et al (2020)
Household cost per neonatal sepsis case	107.80	Okomo et al (2020)
Total health system savings	112 590.60	Estimated/model output
Total household savings	29 998.19	Estimated/model output
Total economic savings	142 588.80	Estimated/model output
Hospital cost per hospitalized LBW case	1721.00	Enweronu-Laryea et al (2020)
Total averted hospital cost	48 870.89	Estimated/model output
Nonmedical household cost per LBW case	52.00	Enweronu-Laryea et al (2018)
Total nonmedical household cost averted	1476.63	Enweronu-Laryea et al (2018)
Medical OOP cost per LBW case	133.00	Enweronu-Laryea et al (2018)
Total medical OOP household cost averted	3776.77	Enweronu-Laryea et al (2018)
Total household cost averted	5253.41	Estimated/model output
Additional ANC visits	14 353.06	Estimated/model output
Total incremental cost of ANC visits	36 743.84	Estimated/model output
No. of births at health facilities in Zipline areas	70 849.00	Estimated/model output
Unit cost per birth (weighted vaginal/C-section)	34.73	GHS
Incremental cost of births shifted to health facilities	407 588.10	Estimated/model output
Cost per ANC visit	2.56	G-DRG Revised Tariffs 2022 2.0 from the NHIS
Cost per vaginal delivery	24.08	G-DRG Revised Tariffs 2022 2.0 from the NHIS
Cost per C-section delivery	49.41	G-DRG Revised Tariffs 2022 2.0 from the NHIS
OOP cost per ANC visit	6.85	Alatinga et al (2024)
OOP cost per vaginal delivery	4.42	Alatinga et al (2024)
OOP cost per C-section delivery	20.60	Alatinga et al (2024)
Total LMD cost of ANC related products	32 160.43	Estimated/model output
Total LMD cost of ANC + PPH prevention products	45 053.48	Estimated/model output
Total LMD cost of ANC + PPH prevention & treatment products	131 130.20	Estimated/model output

Abbreviations: ANC, antenatal care; DALY, disability-adjusted life-year; LMD, last-mile delivery; NHIS, National Health Insurance Scheme; OOP, out of pocket; PPH, postpartum hemorrhage.

**Averted costs associated with reduced burden of disease**: We accounted only for the immediate care costs of ELBW infants, assuming a 100% hospitalization rate for this group, which has been confirmed with the GHS, and a 2.66% hospitalization rate for the LBW and VLBW infants, as provided for the January to December 2022 period by the Ashanti Regional Hospital. We sourced the healthcare costs for the NHIS and the OOP medical costs for caretakers and indirect, nonmedical costs for caretakers of LBW in Ghana from published literature.^15,16^ It is worth noting that the averted healthcare costs in our analysis reflect only the immediate care of ELBW infants and do not account for the long-term incremental healthcare costs typically associated with LBW infants throughout their lives.^17^

To estimate averted costs from reduced PPH, we classified averted cases as mild or severe using the proportion of severe PPH cases (24.82%) reported for Ghana.^18^ Per-case cost estimates sourced from published literature indicated public hospital excess costs of US $72.90 for mild PPH and US $344.35 for severe PPH under NHIS, and US $99.50 (mild) and US $668.00 (severe) for OOP household expenditures.^19^ We multiplied the number of mild and severe cases averted by these unit costs to estimate the total averted costs for both NHIS and households.

To estimate the economic benefit associated with the reduction in neonatal early onset sepsis, we applied 2024-adjusted cost estimates, which reported average treatment costs of US $404.60 to the health system and US $107.80 to households per case of neonatal sepsis.^20^ This allowed us to estimate both the health and economic impacts of improving delivery location access.

**Cost of additional ANC visits and births at health facilities**: To estimate the health system and household costs associated with increased maternal health service utilization due to the interventions, we encountered the challenge of differentiating between costs borne by the government or health system and those representing the OOP burden on households. This distinction was particularly complex in the context of Ghana’s Free Maternal Health Care Policy, under which these services are officially covered. However, substantial evidence indicates that health facilities frequently impose informal or explicit charges on patients and that these practices are largely attributed to systemic underfunding and delays in reimbursements.^21,22^

For estimating the health system cost, we used the 2022 Ghana Diagnosis-Related Groupings (G-DRG) Revised Tariffs (version 2.0) as a proxy for the reimbursement amounts provided by the NHIS for antenatal care visits and deliveries, including both vaginal and cesarean births. These NHIS reimbursement rates served as a conservative estimate of the value the health system is officially prepared to absorb per service rendered.

To estimate the OOP costs borne by households, we relied on cost estimates drawn from published literature for Ghana.^22^

**Aerial logistics LMD cost**: Zipline stored and delivered over 1.5 million units of products across 299 categories from its GH2 distribution center in Ghana’s Ashanti Region in 2021 and 2022. These included medical supplies for pain management, chronic disease management, antimicrobials, antimalarials, and others. Most flights carried a diverse mix of products, except for those requiring cold or ultracold supply chains, which were typically transported in separate flights.

The primary challenge was to isolate the costs of this specific intervention from Zipline’s overall capital and operational expenses. We then needed to allocate the LMD costs to the 3 intervention categories required for ICER calculations: ANC LMD, PPH prevention LMD, and PPH treatment LMD.

We began by identifying which supplies delivered by Zipline could be reasonably attributed to each of the 3 intervention categories. For ANC, this included nutritional supplements commonly provided during antenatal visits, such as iron and folic acid, as well as vaccines administered during ANC in Ghana, including the diphtheria-tetanus vaccine. For PPH prevention, we included all ANC supplies, in addition to oxytocin injections, which are widely used to prevent postpartum bleeding. For PPH treatment, we included all PPH prevention supplies, along with whole blood and plasma products, which are critical for managing severe cases of PPH. A complete reference list of the included products is provided in the **Online Supplementary Material**.

To estimate the LMD cost per product, we first calculated the average percentage of flight occupancy for each item in 2021 and 2022, taking into account the product’s dimensions and the average number of units per flight. For example, while blood products typically occupied 100% of the payload volume on the flights carrying them, diphtheria-tetanus vaccines and folic acid tablets occupied, on average, 25% and 37% of flight volume, respectively.

We then estimated the cost per flight by analyzing Zipline’s monthly operational expenditures and capital depreciation for the GH2 distribution center in 2021-2022, adjusting all figures to 2024 USD. The average LMD cost per unit was calculated by multiplying the average percentage of flight occupancy by the estimated cost per flight.

Finally, we multiplied the per-unit LMD cost by the total number of units delivered during 2021 and 2022 to estimate the total LMD cost per product. These were then aggregated into the 3 intervention categories, specifically ANC, PPH prevention, and PPH treatment, to generate the respective total LMD costs for each.

It is worth noting that, while the exclusive association between our listed ANC and PPH prevention supplies and their respective categories is relatively straightforward, the allocation of blood products exclusively to PPH treatment is less clear. Blood products are also used in the management of trauma cases and surgical emergencies. As a result, we are likely overestimating the LMD costs specific to PPH treatment, since some of the transported supplies serve multiple clinical purposes beyond obstetric care.

To estimate the incremental cost of LMD via aerial logistics, we considered 2 primary pathways through which the intervention could influence maternal and newborn health outcomes. The first was expanded access, where facilities receive ANC and PPH supplies they would not have otherwise obtained. The second was improved delivery efficiency, where supplies that would have eventually arrived through traditional means are delivered more quickly, thereby reducing missed opportunities for treatment and increasing uptake of preventive services. Anecdotal evidence suggests that both mechanisms are at play. This distinction is important for interpreting cost-effectiveness results: when aerial logistics expands access, it adds to existing government supply chain costs; when it simply replaces delayed deliveries, it may not represent a true additional cost. However, due to limited data on stock levels and supply gaps at health facilities, it is not possible to quantify the proportion of deliveries that were additional vs substitutive. To address this uncertainty, the primary ICER analysis adopted a conservative approach by treating all aerial deliveries as incremental costs. While this may overestimate the financial burden, it strengthens the credibility of the findings given the inconsistencies and limited transparency in traditional LMD data systems.

## RESULTS

### Base Case

Under the base-case scenario summarized in **Table 3**, the intervention incurred a total cost of US $444 331.95 for the NHIS, driven by increased use of ANC services and facility-based deliveries. Aerial logistics added US $131 130.23 in storage and LMD costs. These logistics costs were offset by US $174 475.62 in averted government healthcare expenditures due to reduced maternal and newborn illness, with the intervention finally resulting in a net incremental cost of US $400 986.56 from the government perspective. This yielded an ICER of US $106.79 per DALY averted, based on 3754.99 discounted DALYs.

**Table 3.** Base Case Results

**Parameter**	**Value**
Total discounted DALYs averted	3754.99
Total life years saved (undiscounted)	7554.00
Total premature deaths averted	130.49
Total averted healthcare costs (NHIS)	$174 476
Total averted household costs	$57 574
Additional cost for Zipline LMD for maternal health	$131 130
Cost of increased service demand (ANC + births)	$444 332
Additional OOP expenditure in ANC and facility deliveries	$1 075 301
Cost per additional ANC user	$88.46
Cost per additional ANC visit achieved	$2.24
Cost per additional birth at health facility	$5.60
Cost per PPH case averted	$5275
Cost per death averted	$3073
Cost per life year saved	$53.08
Cost per discounted DALY averted (health system perspective)	$106.79
Cost per discounted DALY averted (societal perspective)	$377.82

Abbreviations: ANC, antenatal care; DALY, disability-adjusted life-year; LMD, last-mile delivery; NHIS, National Health Insurance Scheme; OOP, out of pocket; PPH, postpartum hemorrhage.

From a societal perspective, incorporating US $1 075 300.83 in increased OOP spending and US $57 573.81 in household cost savings, the ICER was US $377.82 per DALY averted.

The intervention was highly cost-effective using the threshold of 1 × GDP per capita for Ghana in 2024 (US $2409). When compared with established country-specific cost-effectiveness thresholds (US $126.71-$1158.70),^23^ the intervention remained highly cost-effective from the government perspective and cost-effective from the societal perspective.

The intervention was estimated to prevent approximately 130 premature deaths, at a cost of US $3072.87 per death averted. Access to maternal services improved, with costs of US $88.46 per additional ANC user, US $2.24 per incremental ANC visit, and US $5.60 per additional facility-based delivery.

### One-way Sensitivity Analysis and Tornado Diagram

**Figure 2.**
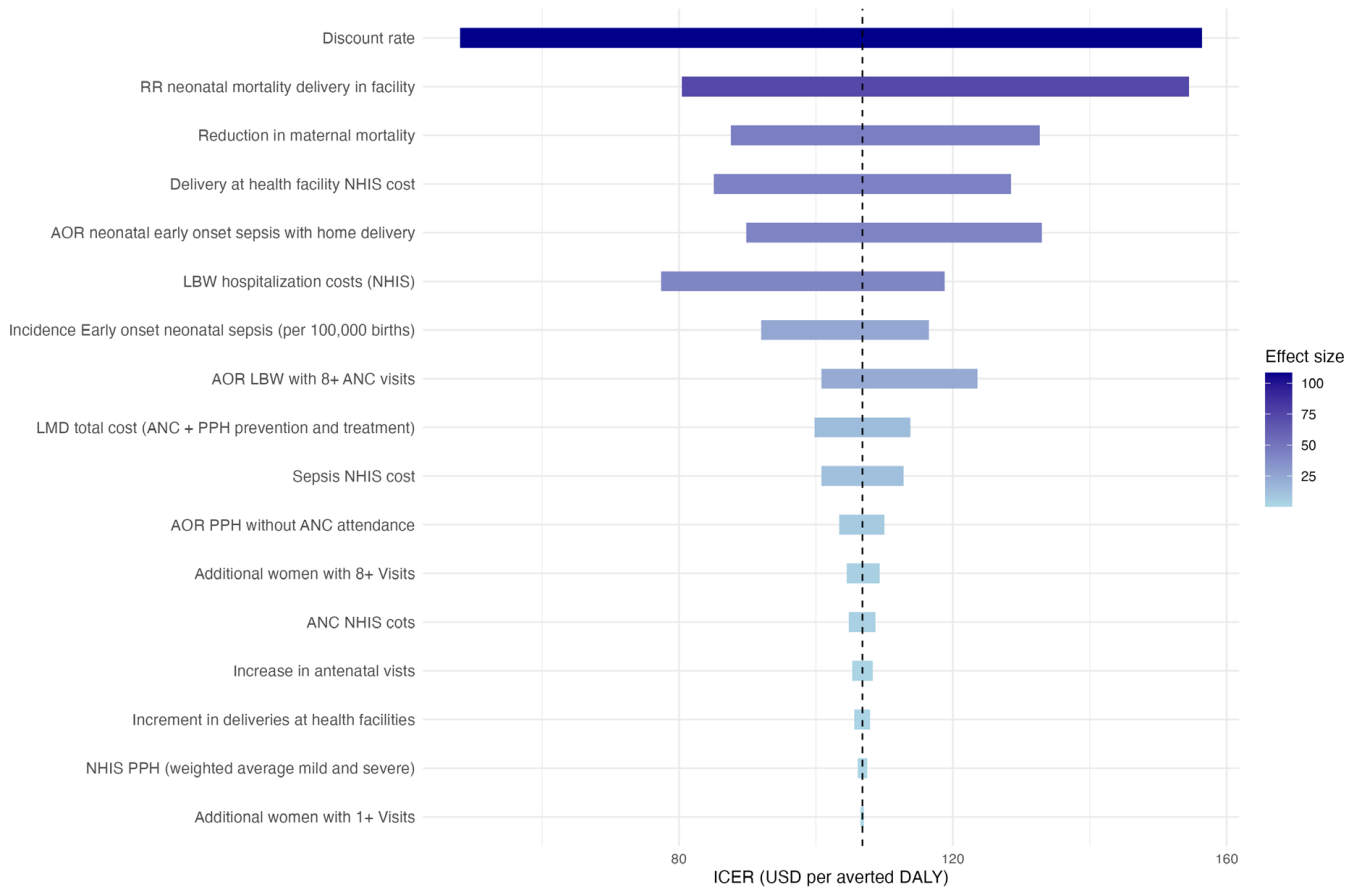
Tornado Plot: Cost Per Averted DALY (Health System Perspective) Abbreviations: ANC, antenatal care; AOR, adjusted odds ratio; DALY, disability-adjusted life-year; LBW, low birth weight; LMD, last-mile delivery; NHIS, National Health Insurance Scheme; OOP, out of pocket; PPH, postpartum hemorrhage; RR, relative risk. This figure displays the results of one-way sensitivity analyses, showing how variations in key parameters influence the ICER (USD per averted DALY) for the aerial logistics intervention, from the healthcare system perspective. Bars represent the change in ICER when each parameter is varied across its plausible range, with longer bars indicating greater influence. Parameters are ordered by effect size, with color intensity also reflecting relative magnitude. The vertical dashed line represents the base-case ICER.

Tornado diagram results (**Figure 2**) identify the discount rate as the most influential parameter on the ICER from the health system perspective, reflecting the importance of how future health gains are valued. Maternal and neonatal mortality assumptions and health service cost inputs also showed substantial impact. Across all tested variations, ICERs remained below conventional willingness-to-pay thresholds, indicating robust cost-effectiveness. The full range of parameter values used in the sensitivity analysis is detailed in the **Supplementary Material**.

From the societal perspective, the ICER ranged from approximately US $300 to US $520 per DALY averted, with a central estimate of US $377.82. Although more sensitive to input variation, the societal ICER remained consistently below the World Health Organization (WHO) threshold. The tornado diagram from the societal perspective is included in the **Supplementary Material**.

## Probabilistic Sensitivity Analysis

Monte Carlo simulations (n = 1000) assessed joint uncertainty across all key parameters, with both cost-effectiveness planes (health system and societal perspectives) presented in the **Supplementary Material**. All ICERs from both perspectives fell below the 1×GDP per capita threshold of US $2409, demonstrating a 100% probability of cost- effectiveness under this benchmark. The probability distributions assigned to each parameter and the corresponding uncertainty ranges used in the analysis are specified in the **Supplementary Material**.

**Figure 3.**
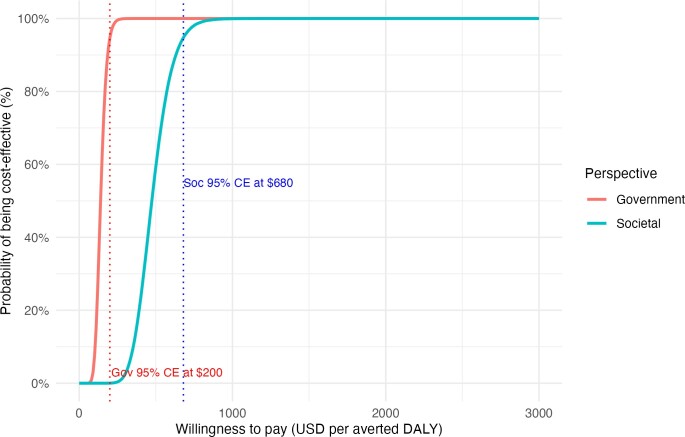
Cost-Effectiveness Acceptability Curves Abbreviations: CE, cost-effectiveness; DALY, disability-adjusted life-year; USD, US dollar. This figure shows the probability that the aerial logistics intervention is cost-effective at varying willingness-to-pay (WTP) thresholds per averted DALY, from both the government or health system perspective (red) and societal (blue) perspectives. The cost-effectiveness acceptability curves are derived from probabilistic sensitivity analysis. Vertical dotted lines indicate the WTP thresholds at which the intervention reaches 95% probability of being cost-effective: $200 under the government perspective and $680 under the societal perspective. The curves illustrate the higher certainty of cost-effectiveness under broader societal valuation.

The cost-effectiveness acceptability curves depicted in **Figure 3** showed a 95% probability of cost-effectiveness at a willingness-to-pay of US $200 (healthcare system perspective) and US $680 (societal perspective). Both curves plateaued at 100% well below the WHO-recommended threshold, confirming strong confidence in the intervention’s economic value.

## DISCUSSION

This analysis demonstrates that aerial logistics is a highly cost-effective strategy for improving MNH outcomes, with ICERs of US $106.79 (government) and US $377.82 (societal) per DALY averted, both below the WHO threshold of US $2409 and within the range proposed by Woods et al. These findings remain robust under both one-way and probabilistic sensitivity analyses, with key drivers including discount rate, mortality reductions, and health service cost inputs.

When benchmarked against other MNH interventions, aerial logistics compares favorably. In Ghana, facility-based quality improvement efforts have reported ICERs of US $178 to $205,^24,25^ while community-level strategies like home visits and emergency transport show higher ICERs, often exceeding US $500.^26^ Only low-dose, high-frequency basic emergency obstetric and newborn care training achieved lower ICER at US $67 per DALY averted.^27^

Across the region, systematic reviews have found that most MNH interventions fall within a cost range of approximately US $150 to $1000 per DALY averted.^28^ Reported estimates include US $82 per DALY averted for insecticide-treated net (ITN) distribution during ANC visits in the DRC, and up to US $406 for quality improvement collaboratives in Niger.^29^ Among these, only ITN distribution, already included in Ghana’s standard MNH package, was more cost-effective than aerial logistics.

This positions aerial logistics as a relatively efficient and scalable intervention. Unlike most MNH strategies focused on direct service delivery, it addresses supply chain bottlenecks by ensuring timely availability of maternal health commodities, and through this mechanism it improves provider readiness while facilitating and incentivizing service use—indirectly influencing outcomes through improved access and continuity of care. These effects can be conceptually separated into logistical, clinical, and behavioral components. Logistically, drone delivery helps ensure timely and reliable access to critical ANC commodities at remote facilities. Clinically, this enables health workers to deliver appropriate screenings and treatments without interruption. Behaviorally, consistent service availability may increase trust in the health system and encourage more women to initiate and complete recommended ANC schedules.

The higher ICER from the societal perspective underscores a persistent policy gap: household OOP expenditures remain substantial despite NHIS coverage mandates.^22^ The intervention’s effectiveness, even with these financial burdens, suggests that supply-side and systemic barriers, such as geographic access and commodity availability, remain critical. Aerial logistics may help address these constraints, particularly in underserved or remote settings.

Three limitations merit consideration. First, in the absence of empirical data on ANC visit distributions by exposure, microsimulation was used to estimate thresholds. Given the stability of Ghana ANC distributions across years, this assumption is reasonable and supported by sensitivity analyses. Second, the cost of aerial logistics was treated as fully incremental (ie, not offsetting any of the baseline due to limited data on conventional LMD). While conservative, this approach likely underestimates cost-effectiveness. Notably, drone systems offer transparent, real-time delivery data, an operational advantage over traditional methods.

Finally, the model does not capture potential economies of scale. Evidence from Ghana suggests that a 10% increase in ANC uptake can reduce unit costs by over 30%, and similar effects are seen for facility deliveries.^30^ Incorporating such dynamics may further improve the cost-effectiveness profile. Future studies should explore how supply chain improvements interact with health system efficiency and long-term delivery costs.

This study adds to the emerging literature on drone-based public health logistics by being the first, to our knowledge, to evaluate the cost-effectiveness of centralization and aerial delivery for routine MNH commodities in a low-resource setting. It extends prior applications focused on emergency obstetric care^31^ and immunization,^32^ providing new evidence that aerial logistics can support broader, routine service delivery and contribute meaningfully to national health goals.

## CONCLUSIONS

Aerial logistics represents a cost-effective, system-level approach to improving MNH outcomes in Ghana. It compares favorably with both facility-based and community-based interventions, including those already implemented nationally. The intervention effectively addresses nonfinancial barriers by improving supply chain responsiveness, and it remains efficient even when accounting for household OOP expenditures. Integrating aerial logistics with policies aimed at financial protection could enhance equity, efficiency, and health outcomes. Future research should assess its long-term scalability, potential cost savings, and broader health system impacts.

## Supplementary Material

Online Supplementary Material
