# Supplementary material for: Cost-Effectiveness of Aerial Logistics for Maternal and Newborn Health: A Simulation-Based Analysis Grounded in Real-World Evidence from the Ashanti Region in Ghana
Source: J Health Econ Outcomes Res. 2025 Sep 17;12(2):143065. doi: 10.36469/001c.143065 (PMC12448434; doi:10.36469/001c.143065)
Supplement: Online Supplementary Material [file jheor_2025_12_2_143065_299202.pdf]

Journal of Health Economics and Outcomes Research

Online Supplementary Material

Cost-Effectiveness of Aerial Logistics for Maternal and Newborn Health: A Simulation-Based Analysis Grounded in Real-World Evidence from the Ashanti Region in Ghana. *JHEOR*. 2025;12(2):??-??.
doi:10.36469/jheor.2025.143065

Table S1: List of Products Included in the Cost of Last-Mile Delivery

Table S2: Parameters Used for One-Way Sensitivity Analysis

Figure S1: Tornado Plot: Cost per Averted DALY (Societal Perspective)

Figure S2: Cost Effectiveness Plane (Health System Perspective)

Figure S3: Cost Effectiveness Plane (Societal Perspective)

This supplementary material has been provided by the authors to give readers additional information about their work.

This is an open-access article distributed under the terms of the Creative Commons Attribution 4.0 International License (CCBY-4.0). View this license's legal deed at http://creativecommons.org/licenses/by/4.0 and legal code at http://creativecommons.org/licenses/by/4.0/legalcode for more information.

Table S1. List of Products Included in the Cost of LMD

ANC products
Iron (III) polymaltose complex capsule
Prenatal multivitamin
Folic acid tablet
Tab folic acid (Birem)
Diphtheria & tetanus ASH
Diphtheria tetanus vaccine (TD)
Mebendazole tablets
Prevention of PPH and management of hypertensive disorders
Oxytocin injection
Magnesium sulfate injection
PPH treatment
WB AB+ adult
WB O- adult
WB B- adult
WB B+ adult
WB A+ adult
WB A- adult
WB O+ adult
WB AB- adult
FFP B+ adult
FFP AB- adult
FFP O- adult
FFP B- adult
FFP A+ adult
FFP O+ adult
FFP AB+ adult
FFP A- adult

Table S2. Parameters Used for One-Way Sensitivity Analysis

Variable	Lower Bound	Central Value	Upper Bound	Source
Increase in antenatal visits	18%	20%	22%	Estimated plausible ranges with ± 20%
Increment in deliveries at health facilities	24%	26%	28%	Estimated plausible ranges with ± 20%
Reduction in maternal deaths	27%	56%	70%	Estimated plausible ranges with ± 20%
Additional women with 8+ visits	2069	2252	2420	Most extreme scenarios from the 1000 simulations
Additional women with 1+ visits	339	364	390	Most extreme scenarios from the 1000 simulations
AOR LBW with 8+ ANC visits	0.602	0.249	0.103	https://pubmed.ncbi.nlm.nih.gov/37644518/
RR neonatal mortality delivery in facility	0.87	0.71	0.54	Tura et al. *BMC Pregnancy Childbirth.* 2013;13:18.
AOR PPH without ANC attendance	1.12	3.43	10.05	https://www.imrpress.com/journal/CEOG/51/10/10.31083/j.ceog5110229/htm
AOR neonatal early onset sepsis with home delivery	1.15	2.67	4	https://pubmed.ncbi.nlm.nih.gov/32997150/
Incidence Early onset neonatal sepsis (per 100,000 births)	946	1412	2097	Based on https://adc.bmj.com/content/106/8/745 and https://pubmed.ncbi.nlm.nih.gov/36141932/
LBW hospitalization costs (NHIS)	$129	$1721	$5 609	https://www.ajol.info/index.php/gmj/article/view/192112
OOP PPH (weighted average mild and severe)	$192.5	$240.0	$288.7	Estimated plausible ranges with ± 20%
NHIS PPH (weighted average mild and severe)	$112.2	$140.2	$288.7	Estimated plausible ranges with ± 20%
ANC NHIS costs	$2.1	$2.6	$3.1	Estimated plausible ranges with ± 20%
ANC OOP cost (including nonmedical)	$5.5	$6.8	$8.2	Estimated plausible ranges with ± 20%
Delivery at health facility NHIS cost	$22.3	$27.9	$33.4	Estimated plausible ranges with ± 20%
Delivery at health facility OOP cost (including nonmedical)	$53.5	$66.8	$80.2	Estimated plausible ranges with ± 20%
Sepsis NHIS cost	$323.7	$404.6	$485.5	Estimated plausible ranges with ± 20%
Sepsis OOP cost	$86.2	$107.8	$129.4	Estimated plausible ranges with ± 20%
LMD total cost (ANC + PPH prevention and treatment)	$104 904.0	$131 130.0	$157 356.0	Estimated plausible ranges with ± 20%
Discount rate	0%	3%	5%	Recommended range by WHO

Abbreviations: ANC, antenatal care; AOR, adjusted odds ratio; LBW, low birthweight; RR, risk ratio; PPH, postpartum hemorrhage; NHIS, National Health Insurance Scheme; OOP, out-of-pocket; LMD, last-mile delivery; USD, US dollar; WHO, World Health Organization.

Figure S1. Tornado Plot: Cost per Averted DALY (Societal Perspective)

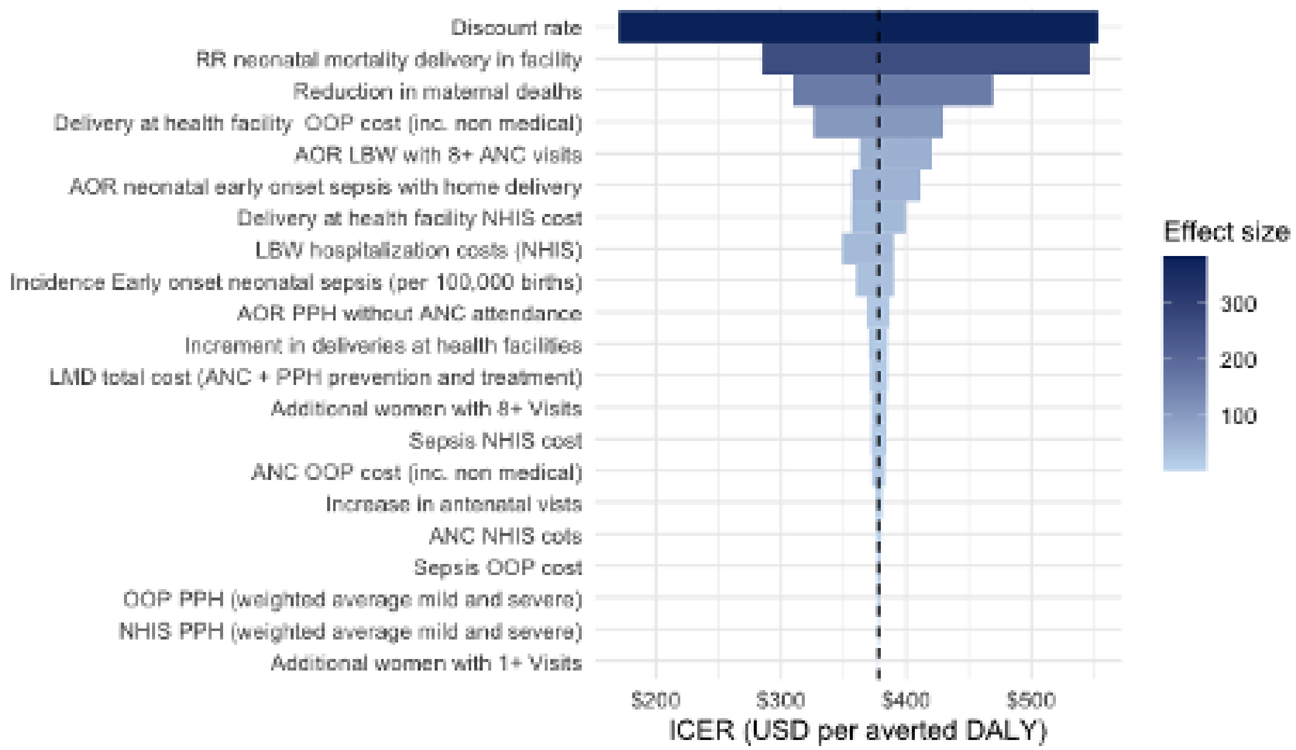

Abbreviations: ICER, incremental cost-effectiveness ratio; DALY, disability-adjusted life-year; USD, US dollar; RR, risk ratio; AOR, adjusted odds ratio; ANC, antenatal care; LBW, low birthweight; PPH, postpartum hemorrhage; NHIS, National Health Insurance Scheme; OOP, out-of-pocket; LMD, last-mile delivery.

Figure S2. Cost Effectiveness Plane (Health System Perspective)

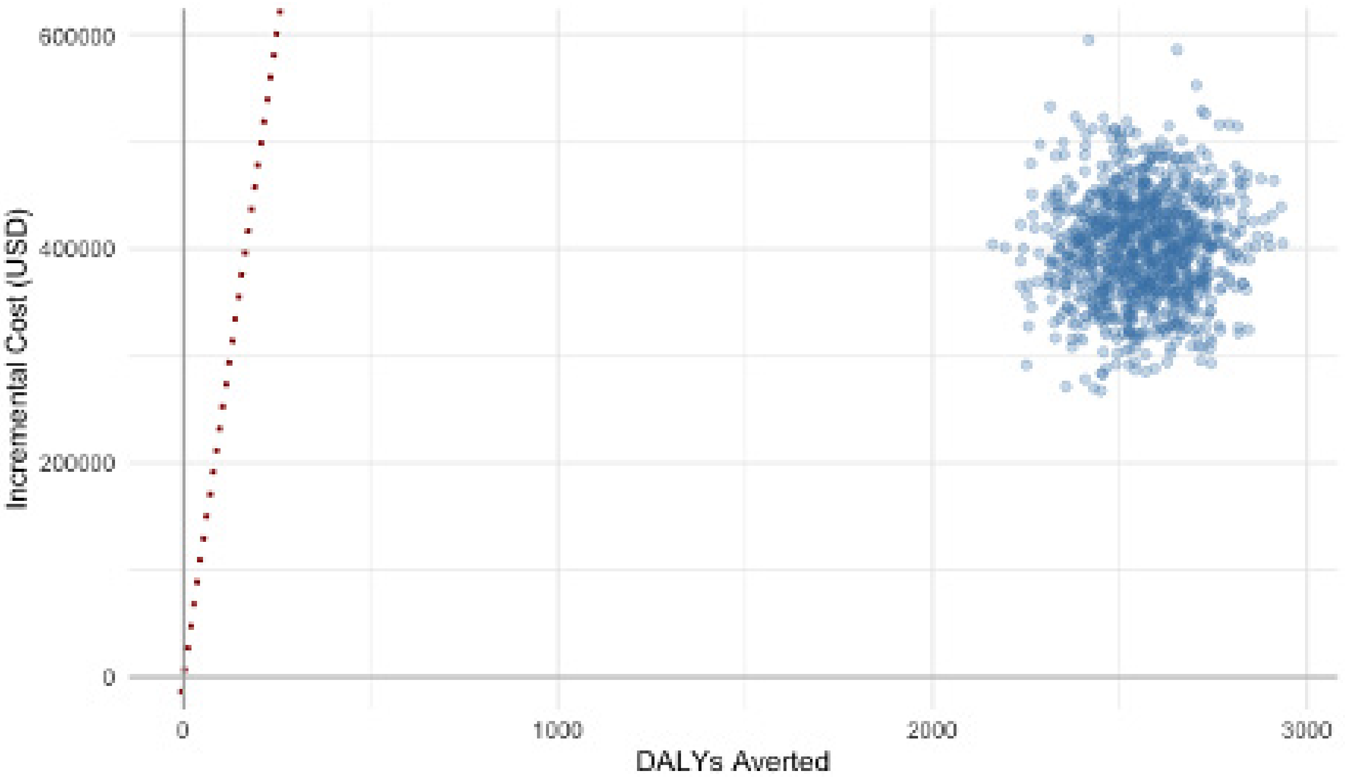

Abbreviations: DALY, disability-adjusted life-year; USD, US dollar.

Figure S3. Cost Effectiveness Plane (Societal Perspective)

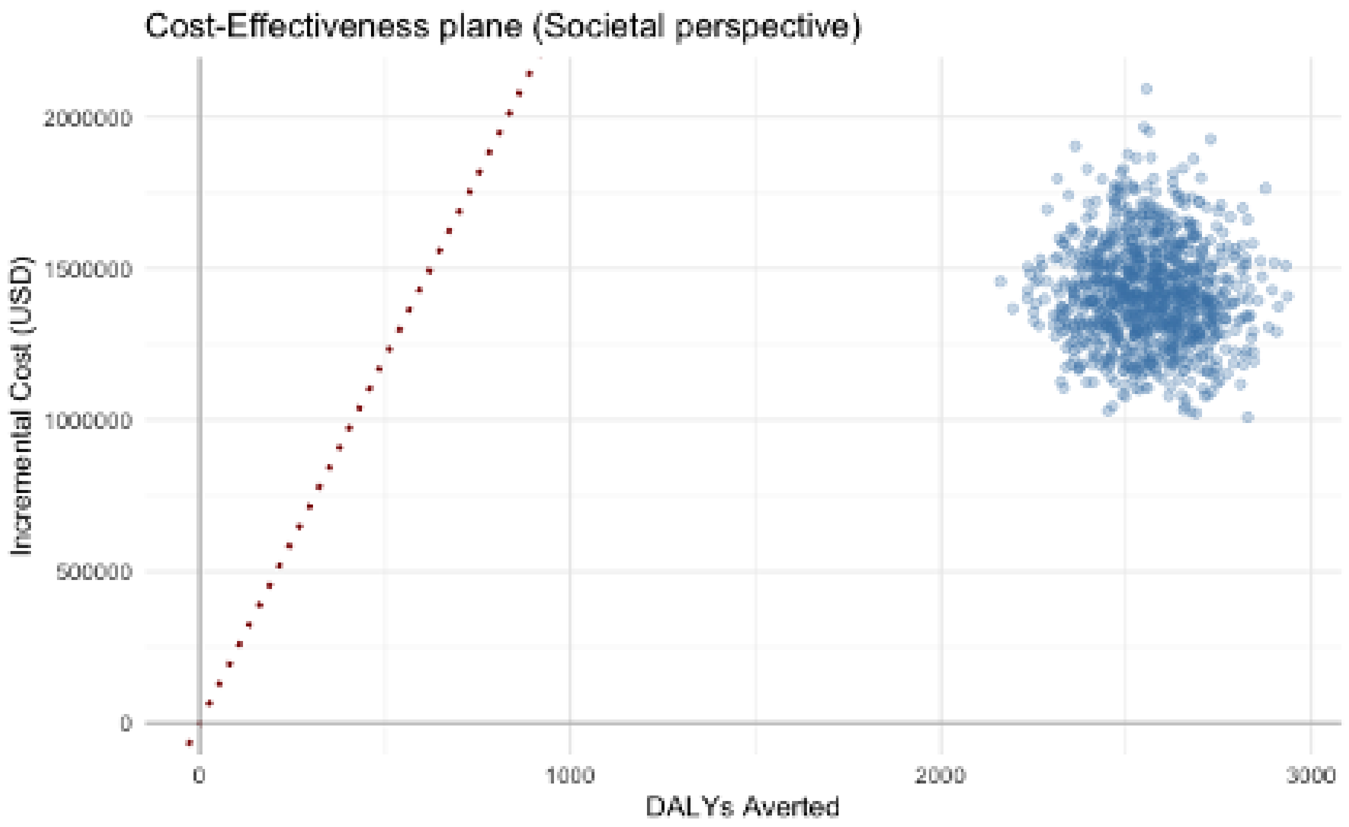

Abbreviations: DALY, disability-adjusted life-year; USD, US dollar.
